# Boreal predator co‐occurrences reveal shared use of seismic lines in a working landscape

**DOI:** 10.1002/ece3.6028

**Published:** 2020-01-30

**Authors:** Erin R. Tattersall, Joanna M. Burgar, Jason T. Fisher, A. Cole Burton

**Affiliations:** ^1^ Department of Forest Resources Management University of British Columbia Vancouver Canada; ^2^ School of Environmental Studies University of Victoria Victoria Canada; ^3^ Ecosystems Management Unit InnoTech Alberta Victoria Canada

**Keywords:** camera traps, community ecology, facilitation, large carnivores, predator interactions

## Abstract

Interspecific interactions are an integral aspect of ecosystem functioning that may be disrupted in an increasingly anthropocentric world. Industrial landscape change creates a novel playing field on which these interactions take place, and a key question for wildlife managers is whether and how species are able to coexist in such working landscapes. Using camera traps deployed in northern Alberta, we surveyed boreal predators to determine whether interspecific interactions affected occurrences of black bears (*Ursus americanus*), coyotes (*Canis latrans*), and lynx (*Lynx canadensis*) within a landscape disturbed by networks of seismic lines (corridors cut for seismic exploration of oil and gas reserves). We tested hypotheses of species interactions across one spatial‐only and two spatiotemporal (daily and weekly) scales. Specifically, we hypothesized that (1) predators avoid competition with the apex predator, gray wolf (*Canis lupus*), (2) they avoid competition with each other as intraguild competitors, and (3) they overlap with their prey. All three predators overlapped with wolves on at least one scale, although models at the daily and weekly scale had substantial unexplained variance. None of the predators showed avoidance of intraguild competitors or overlap with prey. These results show patterns in predator space use that are consistent with both facilitative interactions or shared responses to unmeasured ecological cues. Our study provides insight into how predator species use the working boreal landscape in relation to each other, and highlights that predator management may indirectly influence multiple species through their interactions.

## Graphical abstract

Interspecific interactions are an integral aspect of ecosystem functioning that may be disrupted in an increasingly anthropocentric world. Using camera traps deployed in northern Alberta, we surveyed boreal predators to determine whether interspecific interactions affected occurrences of black bears (*Ursus americanus*), coyotes (*Canis latrans*), and lynx (*Lynx canadensis*) within a seismic line network. Our results highlight important considerations of the impact of predator management decisions, especially in working landscapes shared between wildlife and industry.

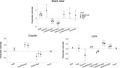

## 1 INTRODUCTION

Human resource consumption is driving dramatic changes to natural landscapes around the world (MacDougall, McCann, Gellner, & Turkington, 2013; World Wildlife Fund, 2018). These changes alter the suitability of landscapes in different ways for different species, which in turn is likely to alter the interactions among these species (e.g., Steinmetz, Seuaturien, & Chutipong, 2013). Interspecific interactions are an integral aspect of ecosystem function, as they influence both population dynamics of interacting species and community‐level responses to environmental change (MacMahon, Phillips, Robinson, & Schimpf, 1978). Of the many types of interactions described in classic ecological theory, two important types are facilitation—in which one species benefits from another (Bertness & Callaway, 1994; Bruno, Stachowicz, & Bertness, 2003)—and competition—in which one species dominates another (Alley, 1982; Schoener, 1974). Although consequences for interacting species vary, both facilitation and competition can influence resource availability (Bruno et al., 2003; Wiens, 1993) and thus ultimately species coexistence (MacMahon et al., 1978). Maintaining these interactions is therefore vital to maintaining resilient ecological communities in an increasingly anthropocentric world (Valiente‐Banuet et al., 2015).

The Canadian boreal forest is one biome experiencing dramatic landscape change due to natural resource extraction, which is creating a heterogeneous “working” landscape shared between industry and wildlife (Pickell, Andison, Coops, Gergel, & Marshall, 2015). Logging alone has an area footprint of approximately 15 million hectares, while energy development has created over half a million kilometers of linear features across the region (Pasher, Seed, & Duffe, 2013). The cumulative effects of these extensive industrial footprints impact the distribution and abundance of a number of boreal mammals, though the strength and nature of influence vary by species (Fisher & Burton, 2018; Toews, Juanes, & Burton, 2018). Behavioral and population changes in individual species can in turn affect interactions among species (Ritchie & Johnson, 2009), leading to broader indirect effects of industrial development in boreal ecosystems.

In northeastern Alberta, where industrial development has the greatest footprint (Pickell et al., 2015), linear features directly affect animal behaviors and population dynamics. Networks of seismic lines—wide trails (5 m–10 m, Dabros, Pyper, & Castilla, 2018) cut for seismic exploration of oil and gas reserves—facilitate movement for gray wolves (*Canis lupus*) across challenging boreal wetland terrain (Dickie, Serrouya, McNay, & Boutin, 2016), improving their abilities to hunt in these habitats (McKenzie, Merrill, Spiteri, & Lewis, 2012). Like wolves, evidence suggests that black bears prefer linear features for ease of travel (Latham, Latham, & Boyce, 2011; Tigner, Bayne, & Boutin, 2014). Mesocarnivores such as coyotes (*Canis latrans*) and Canada lynx (*Lynx canadensis*) may also benefit from linear features. Human development, including linear corridors, is positively related to coyotes' northern expansion and persistence in the boreal forest (Hody & Kays, 2018) and lynx presence (Fisher & Burton, 2018; but see Bayne, Boutin, & Moses, 2008). With at least some members of the boreal predator community responding to extensive seismic line networks, we expect these networks to potentially influence the interactions among predators. More specifically, we predict that linear disturbances result in changes in the co‐occurrence patterns of sympatric predator species. We hypothesize that these changes may be driven by three different types of intraguild interactions.

First, wolves may exert top‐down influences on subordinate predators that influence how the latter use the landscape (Estes et al., 2011). Wolves are commonly regarded as dominant over black bears, coyotes and lynx in direct confrontation, suggesting that these subordinate predators would seek to avoid encounters with wolves (Fuller & Keith, 1981; Palomares & Caro, 1999). However, predators also benefit from scavenging subsidies proffered by wolf kills and may thus have a facilitative interaction with wolves (Allen, Elbroch, Wilmers, & Wittmer, 2014; Atwood & Gese, 2008, 2010; Paquet, 1992; Wilmers, Crabtree, Smith, Murphy, & Getz, 2003). In addition, some mesocarnivores may indirectly profit from wolves via suppression of competitors, as demonstrated for red foxes (*Vulpes vulpes*; Levi & Wilmers, 2012; Sivy, Pozzanghera, Colson, Mumma, & Prugh, 2018) and suggested for lynx (Ripple, Wirsing, Beschta, & Buskirk, 2011).

Second, subordinate predators may experience intraguild competition from one another. Although there is little evidence in the literature of intraguild competition between black bears and other subordinate predators, competition between coyotes and lynx has been insinuated in areas of human disturbance (Bayne et al., 2008) and may influence habitat selection (Murray, Boutin, & O'Donoghue, 1994). Third, all three subordinate predators should track availability of their prey. Black bears predate boreal ungulates (Linnell, Aanes, & Andersen, 1995; Pinard, Dussault, Ouellet, Fortin, & Courtois, 2012). Both coyotes and lynx predate small mammals such as red squirrels (*Tamiasciurus hudsonicus*) and snowshoe hare (*Lepus americanus*), and coyotes also predate white‐tailed deer (Latham, Latham, Boyce, & Boutin, 2013; O'Donoghue et al., 2001). Interspecific interactions are not necessarily mutually exclusive and may happen simultaneously within the same systems, including across separate spatial or temporal scales (Karanth et al., 2017; Sivy, Pozzanghera, Grace, & Prugh, 2017).

In this study, we investigated how apex predators —wolves—and subordinate predators—black bears, coyotes, and lynx—interact on a disturbed landscape by assessing spatiotemporal co‐occurrences within a network of linear anthropogenic features. We developed predictions of co‐occurrence patterns consistent with hypothesized interactions. Specifically, we predicted that (1) if black bears, lynx, and coyotes experience top‐down pressures from wolves, they should spatiotemporally segregate from wolves; (2) subordinate predators should segregate from their intraguild competitors with whom they share resources (O'Donoghue et al., 2001; Guillaumet, Bowman, Thornton, & Murray, 2015); and (3) subordinate predators should overlap with their prey (Keim, DeWitt, & Lele, 2011; Theuerkauf, 2009). We further hypothesized that higher densities of disturbance would result in lower occurrences of black bears, but higher occurrences of lynx and coyotes (Fisher & Burton, 2018), and that coyotes and lynx would occur less frequently in the winter (Pozzanghera, Sivy, Lindberg, & Prugh, 2016).

To test these hypotheses, we modeled black bear, coyote, and lynx spatiotemporal occurrences in relation to wolf and prey occurrences, and coyote and lynx spatiotemporal occurrences relative to one another (Table 1). We further compared the effects of interspecific interactions on predator occurrences with the effects of season and anthropogenic disturbance, allowing for interactions between the two (Table 1).

**Table 1:** Candidate model sets to test the relative effect of interspecific interactions on predator occurrences

Species	Hypothesis—Predator occurrence best explained by	Predictor variables
Mesocarnivore 1	Habitat	Significant forest cover variables from step 1
	Anthropogenic features	Linear density (LD) + Habitat
	Seasonality	Snow + Habitat
	Apex predator	Wolf + Habitat
		Wolf + Snow + Habitat
		Wolf × Snow + Habitat
		Wolf + LD + Habitat
		Wolf × LD + Habitat
	Intraguild competition	Mesocarnivore2 + Habitat
		Mesocarnivore2 + Snow + Habitat
		Mesocarnivore2 × Snow + Habitat
		Mesocarnivore2 + LD + Habitat
		Mesocarnivore2 × LD + Habitat
	Predation opportunities	Prey + Habitat
		Prey + Snow + Habitat
		Prey × Snow + Habitat
		Prey + LD + Habitat
		Prey × LD + Habitat
Black bear	Habitat	Significant forest cover variables from step 1
	Anthropogenic features	LD + Habitat
	Apex predator	Wolf + Habitat
		Wolf + LD + Habitat
		Wolf × LD + Habitat
	Predation opportunities	Prey + Habitat
		Prey + LD + Habitat
		Prey × LD + Habitat

Note

Models were negative binomial GLMs at the spatial‐only scale, and binomial GLMMs at the two spatiotemporal scales. Each model set corresponds to a hypothesized interspecific interaction. We tested models with co‐occurring species as a predictor variable against three base models describing environmental effects. Candidate model sets for mesocarnivores (coyote and lynx) are identical, with mesocarnivore 1 describing the responding predator and mesocarnivore 2 describing the co‐occurring intraguild competitor (e.g., when mesocarnivore 1 is coyote, mesocarnivore 2 is lynx and vice versa). At the spatial‐only scale of analysis, we excluded season models for all species because the response variable aggregated detections across the entire survey period.

## 2 METHODS

### 2.1 Sampling design

**Figure 1:**
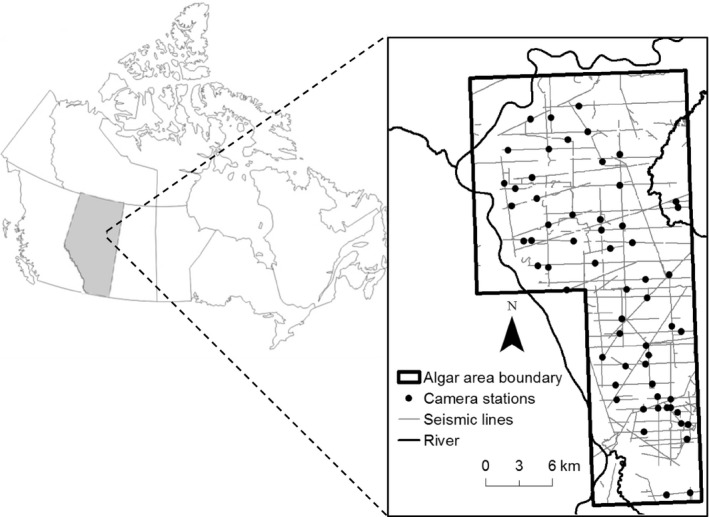
The study area, camera trap locations, and linear disturbances along the east side of the Athabasca River (56.2588 N, 112.6909 W). The inset map shows the location of the study area in Alberta, Canada

Our study was located along the east side of the Athabasca River, approximately 70 km southwest of Fort McMurray, Alberta, Canada (56.2588 N, 112.6909 W; Figure 1). The 570‐km^2^ study area is bounded to the north and west by the Athabasca River and has linear feature density of 1.1 km/km^2^, including 523.6 km of seismic lines (Figure 1; Alberta Biodiversity Monitoring Institute, abmi.ca). We deployed Reconyx PC900 HyperFire camera traps (Reconyx, Holman, WI) between November 2015 and 2016 as part of an ongoing monitoring project assessing wildlife responses to seismic line restoration. One of the objectives of this restoration was to deter predator movements, but the effect on predator line use was minimal (Tattersall, Burgar, Fisher, & Burton, 2019). We selected 60 camera sites (Figure 1) based on a stratified random design to sample along seismic lines that spanned a gradient of restoration strata. We installed one camera at each site at a height of approximately 1 m and facing across a seismic line. Cameras were set up at least 500 m apart to increase the probability of independent detections (Tigner et al., 2014). We set all cameras to take one image per trigger, with a one‐second lag between triggers and no quiet periods.

We considered a survey period as 30 continuous months between November 2015 and April 2018 for coyotes and lynx, and the two eight‐month periods between April and October 2016 and 2017 for black bears. We treated detection events of the same species as independent when occurring at least 30 min apart (Rovero & Zimmermann, 2016). All methods for wildlife monitoring were approved by the Canadian Council of Animal Care administered by the University of British Columbia (protocol A17‐0035).

### 2.2 Analytical framework

For large mammal species ranging across entire landscapes, interspecific interactions can be inferred using spatiotemporal relationships of species occurrences (Cusack et al., 2016; Karanth et al., 2017; Swanson, Arnold, Kosmala, Forester, & Packer, 2016). Correlation is not equivalent to causality, but examining these associations reveals whether patterns in predator co‐occurrences are consistent with those predicted from interspecific interactions.

For interactions between predators, we assumed segregation of occurrences in space or time was suggestive of competition, while overlap in space and time was suggestive of facilitation (Cusack et al., 2016; Fahrig, 1992; Frey, Fisher, Burton, & Volpe, 2017; Karanth et al., 2017). To address multiple potential scales of interactions, we examined predator co‐occurrences at the spatial‐only scale (i.e., entire survey period) and at two finer spatiotemporal scales (weekly and daily). We predicted that spatial segregation was indicative of competition (Fuller & Keith, 1981), whereas spatial overlap alone indicated weak or indirect interactions. We assumed that overlap at finer spatiotemporal scales—where species co‐occur at a given location within a given occasion length—suggested intentional proximity between two species, and thus was evidence of a facilitative interaction (Cusack et al., 2016; Swanson et al., 2016).

#### 2.2.1 Spatial relationships in species' occurrences

To examine spatial relationships between predators, we tested the degree to which spatial variation in species detections across the entire survey (i.e., relative abundance or frequency of use) was explained by the detections of potentially interacting species, using number of independent detections per camera trap site as the response variable. We included each camera's survey effort (number of days active) as a predictor variable, thus accounting for the effect of periods of camera inactivity on detections. This predictor variable was the only fixed effect in the null model and excluded from comparisons of effect size. We modeled detections in a generalized linear model (GLM) framework using a negative binomial distribution for overdispersed count variables (Bolker, 2009). We conducted all statistical analyses using the R package *glmmTMB* (Brooks, Kristensen, & Benthem, 2017).

#### 2.2.2 Fine and coarse spatiotemporal scales of occurrence

To test whether species co‐occurring in space were also co‐occurring in time, we examined co‐occurrences at two temporal scales. At the finest temporal scale, we recorded the presence (1) or absence (0) of a species' at a given site within each day, producing a binary occurrence metric for each camera trap day. Although methods exist for assessing finer scale temporal niche partitioning, they require large amounts of detection data (Frey et al., 2017; Swanson et al., 2016; Wang, Allen, & Wilmers, 2015). Given our observed detection rates, a daily occasion length was the finest temporal scale we could reliably model. However, a single day occasion length results in low detection rates, leading to zero‐inflated occurrence data (Rovero & Zimmermann, 2016). We therefore also discretized occurrence data into week‐long periods to assess whether results were consistent across temporal scales. For both spatiotemporal scales, we modeled occurrences using binomial generalized linear mixed effects models (GLMMs; Cusack et al., 2016). We included a random effect of camera trap site to account for repeated (nonindependent) sampling within sites. We omitted inactive days from the daily occurrence analysis and included the predictor variable *proportion of active days per week* in the weekly occurrence analysis.

### 2.3 Modeling approach

#### 2.3.1 Habitat modeling and scale analysis

We analyzed data using a two‐step process: species‐habitat modeling followed by co‐occurrence modeling (Chow‐Fraser, 2018; Cusack et al., 2016). In the first step, we modeled species response to habitat at multiple spatial scales (Fisher, Anholt, & Volpe, 2011; Levin, 1992). We ranked species‐habitat models at multiple spatial scales using model selection with Akaike's information criterion (AIC, Burnham & Anderson, 2002) to determine the characteristic scale of selection (i.e., scale of best ranked model; Fisher, Anholt, & Volpe, 2011). Each habitat model consisted of six variables describing forest cover types predicted to have an effect on predator occurrence (Latham et al., 2013; Poole, Wakelyn, & Nicklen, 1996; Tigner et al., 2014). We used Alberta Vegetation Inventory digital forest data (AVI; Alberta Vegetation Interpretation Standards 2005) and reclassified by dominant tree species and moisture regime, to create five habitat variables describing forest types (Table 2; Fisher & Burton, 2018). Additionally, we included a predictor variable for the proportion of open forest as a measure of forest density (Murray et al., 1994). We quantified the proportion of each variable around cameras within buffers of 250‐ to 2000‐m radius (spatial scales), at intervals of 250 m. This resulted in eight habitat models, consisting of six habitat variables, one for each spatial scale (and each consisting of the six habitat variables). We used AIC model selection to compare among models and retained the characteristic scale of selection and significant habitat variables of the best supported (lowest AIC) model in the subsequent stage of modeling co‐occurrences (described below; Figure 3a and Figure 4). We extracted all spatial variables using the R packages *rgeos* and *rgdal* (Bivand & Rundel, 2019; Bivand, Keitt, & Rowlingson, 2019).

**Table 2:** Full list of predictor variables used to model occurrence patterns of black bears, lynx, and coyotes

Predictor variables	Step of modeling process	Description
pOpen	1	Proportion of forest with <50% density surrounding camera stations
UpCon	1	Proportion of forest with black spruce (*Picea mariana*), white spruce (*Picea glauca*), balsam fir (*Abies balsamea*), or jack pine (*Pinus banksiana*) as a dominant tree species and a dry or mesic moisture regime
LowCon	1	Proportion of forest with black spruce (*Picea mariana*), white spruce (*Picea glauca*), balsam fir (*Abies balsamea*), or jack pine (*Pinus banksiana*) as a dominant tree species and a wet or aquatic moisture regime
UpDecid	1	Proportion of forest with trembling aspen (*Populus tremuloides*), balsam poplar (*Populus balsamifera*), or paper birch (*Betula papyrifera*) as a dominant tree species and a dry or mesic moisture regime
LowDecid	1	Proportion of forest with trembling aspen (*Populus tremuloides*), balsam poplar (*Populus balsamifera*), or paper birch (*Betula papyrifera*) as a dominant tree species and a wet or aquatic moisture regime
Tamarack	1	Proportion of forest with Tamarack (*Larix laricina*) as a dominant tree species
Wolf	2	Binary presence (1)/ absence (0) of wolves per site per day or week; number of detections of wolves per site
Lynx	2	Binary presence (1)/ absence (0) of lynx per site per day or week; number of detections of lynx per site
Coyote	2	Binary presence (1)/ absence (0) of coyotes per site per day or week; number of detections of coyotes per site
Prey	2	Binary presence (1)/ absence (0) of prey species^1^ per site per day or week; number of detections of prey per site.
LD	2	Linear density measured as total length of linear features divided by a given area surrounding camera stations
Snow	2	Binary presence (1)/ absence (0) of snow per site per day, or number of snow days/ total days in a weekly sampling period. We marked snow as ‘present’ in daily time‐lapse images if it covered 50% of the seismic line surface within the camera's field of view

Note

^1^Prey species consisted of snowshoe hare (*Lepus americanus*) and red squirrel (*Tamiasciurus hudsonicus*) for lynx; hare, squirrel, and white‐tailed deer (*Odocoileus virginianus*) for coyotes; and deer, moose (*Alces alces*), and caribou (*Rangifer tarandus*) for black bears (Latham et al., 2013; Zager & Beecham, 2006; Linnell et al., 1995; O'Donoghue et al., 2001)

For each species, we included all habitat variables in the first step of the modeling process, and retained habitat variables with confidence intervals that did not overlap zero to create a null model for the second step. We measured forest cover variables from the Alberta Vegetation Inventory (Alberta Vegetation Interpretation Standards, 2005) and linear feature data from the Alberta Biodiversity Monitoring Institute (ABMI, abmi.ca). We used camera trap data to extract all species occurrence and snow variables. Species' variables differed with modeling scale, as spatiotemporal scale affected occasion length and thus occurrence aggregation.

#### 2.3.2 Co‐occurrence modeling

In the second step of our two‐step approach, we added heterospecific occurrences as variables to the best supported habitat model and weighed evidence for their ability to explain additional variation in predator occurrences (Table 1; Fisher et al., 2013). We created a model set for each of the interactions hypothesized to influence black bears, coyotes, and lynx, namely top‐down influences from wolves, bottom‐up influences from prey species, or competitive influences of lynx and coyotes on each other. We excluded models assessing interspecific interactions between black bears and coyotes or lynx because we found no supporting evidence for these interactions in the literature (Table 1). To assess influences of prey, we included a variable aggregating detections of all prey species for each of the target predators (Table 2). As we further predicted that the strength of interspecific interactions could be influenced by season and level of anthropogenic disturbances, we also included additive and interaction models with variables for snow presence and linear density (Table 1). Linear features are the most prevalent anthropogenic disturbance within the study area (Tattersall et al., 2019); therefore, we considered the effects of other anthropogenic features to be negligible. We tested all co‐occurrence models against three base models (i.e., models without species interactions): one with only habitat variables, one with snow presence and habitat, and one with linear density and habitat. Because black bears are inactive in the winter, we only included snow presence in model sets for lynx and coyotes. At the spatial‐only scale, we excluded season from the analysis for all species because the response variable aggregated detections across the entire survey period.

We assessed snow presence from daily “time‐lapse” images, using the camera trapping software Timelapse 2.0 Image Analyzer (Greenberg & Godin, 2015; http://saul.cpsc.ucalgary.ca/timelapse). Snow was measured as a binary variable at the daily scale and a proportion at the weekly scale (i.e., mean number of days on which snow was present; Table 1). We considered snow to be present if it covered over 50% of the line surface within the camera's field of view. As with forest cover variables, we conducted a scale analysis to obtain appropriate scales of measurement for linear density for each species (Figure 3b). We used data exploration techniques prior to modeling to assess all predictor variables for outliers, collinearities, and heterogeneity of variance (Zuur, Ieno, & Elphick, 2010). We also scaled all nonbinary variables by subtracting the mean and dividing by two standard deviations, thus improving model convergence and interpretation (Gelman, 2008).

### 2.4 Model interpretation and model validation

Following co‐occurrence modeling, we compared candidate models using AIC model selection to determine whether interspecific interactions influenced predator occurrences at each of the three spatiotemporal scales. We considered all models within 2ΔAIC of the top‐ranked model as having similar explanatory power over the data (Burnham & Anderson, 2002). We consequently examined variables in these models for their influence on predator occurrence, with mean parameter estimates as measures of effect size and direction and 95% confidence intervals as measures of statistical significance (i.e., not overlapping zero). We used pseudo‐R^2^ to assess the proportion of variance explained by the top models for each species relative to the proportion of variance explained by a null model (McFadden, 1974). We considered R^2^ values between 0.2 and 0.4 to be indicative of good model fit (McFadden, 1977).

In both top‐ranked models for coyotes and lynx at the daily scale, standard errors around the estimates for interacting species were over three orders of magnitude larger than the estimates (−15.730 [−7943, 7912] for the top coyote model and −15.763 [−8613, 8582] for the top lynx model), and patterns in the model residuals indicated model misspecification. We therefore removed these models from all subsequent analyses.

## 3 RESULTS

From November 2015 to April 2018, total sampling effort was 14,054 camera‐days for black bears (during their active season) and 32,436 camera‐days for all other species (year‐round). Of the four focal boreal predator species, black bears and wolves were detected most frequently, followed by coyotes and lynx (Table 3). At the daily spatiotemporal scale, both coyotes and lynx infrequently co‐occurred with interacting species, and lynx infrequently co‐occurred with their prey at the weekly scale (Table 3). For all species, models at the daily and weekly scales explained relatively little variance in occurrence data (pseudo‐*R* ^2^ = 0.008–0.074), while models at the spatial‐only scale performed much better (pseudo‐*R* ^2^ = 0.166–0.283; Table 4).

**Table 3:** Total co‐occurrences of predator species across three spatiotemporal scales of analysis

Spatiotemporal scale		Wolf	Prey	Black bear	Lynx	Coyote
Day	Black bear	15	16		–	–
	Lynx	2	1	–		2
	Coyote	2	8	–	2	
	Total	179/295	–	315	71	131
Week	Black bear	33	55		–	–
	Lynx	5	2	–		6
	Coyote	15	22	–	6	
	Total	124/224	–	226	67	106
Spatial‐only	Black bear	38	43		–	–
	Lynx	23	18	–		17
	Coyote	21	18	–	17	
	Total occupied sites	46	–	44	27	23
	Total detections (across sites)	334	–	360	73	154

Note

Each value represents the total number of times both species were present at the same site and—for weekly and daily scales—within the same occasion. Rows represent response variables, and columns represent predictor variables. The total occurrences of wolves are given both within the summer‐only sampling period for black bears (14,054 site‐days) and the full sampling period for coyotes and lynx (32,436 site‐days). Cells are marked with a dash where no interactions were hypothesized or tested.

**Table 4:** Model selection tables of models of top‐ranked models for black bears, coyotes, and lynx

Species	Scale	Predictor variables	*k*	ΔAIC	AICwt	*R* ^2^
Black bear	Day	Wolf + LD +pOpen	5	0.00	0.510	0.00818
		Wolf × LD + pOpen	6	1.66	0.223	0.00805
	Week	Wolf + LD +pOpen	6	0.00	0.533	0.0196
		Wolf × LD + pOpen	7	1.23	0.289	0.0201
	Spatial‐only	Wolf × LD + pOpen +UpCon + LowCon +Tamarack	10	0.00	0.752	0.166
Coyote	Day	Lynx × season + pOpen	6	0.00	0.373	0.0513
		Season + pOpen	4	1.16	0.209	0.0475
		Lynx + season +pOpen	5	1.28	0.196	0.0489
	Week	Wolf + LD +pOpen	6	0.00	0.420	0.0561
		Wolf × LD + pOpen	7	1.25	0.225	0.0571
		Lynx × LD + pOpen	7	1.53	0.195	0.0567
	Spatial‐only	Wolf + LD +pOpen	6	0.00	0.270	0.241
		LD + pOpen	5	0.47	0.214	0.228
		Wolf × LD + pOpen	7	0.78	0.183	0.247
Lynx	Day	Coyote × season + pOpen +LowCon + UpCon	8	0.00	0.220	0.0554
		Season + pOpen +LowCon + UpCon	6	0.26	0.193	0.0510
		Coyote + season +pOpen + LowCon +UpCon	7	0.34	0.186	0.0530
		Wolf + season +pOpen + LowCon +UpCon	7	1.21	0.121	0.0521
		Wolf × season + pOpen +LowCon + UpCon	8	1.36	0.112	0.0540
	Week	Prey × LD + pOpen +LowCon + UpCon	9	0.00	0.597	0.0744
	Spatial‐only	Wolf + LD+pOpen + LowCon +UpCon	8	0.00	0.360	0.279
		Prey × LD + pOpen +LowCon + UpCon	9	1.24	0.194	0.283
		Wolf × LD + pOpen +LowCon + UpCon	9	1.48	0.172	0.282

Note

For each species, top‐ranked models are shown for each of the three spatiotemporal scales of analysis. Top‐ranked models were those within 2ΔAIC of the highest weighted model. The column *k* is the number of parameters in the model, ΔAIC indicates the difference in AIC scores from the top model, and *R* ^2^ is a pseudo‐*R* ^2^ measure describing the proportion of variance explained by each model relative to the variance explained in the null model. The top models for coyote and lynx at the daily scale were not included in subsequent analyses due to large confidence intervals.

**Figure 2:**
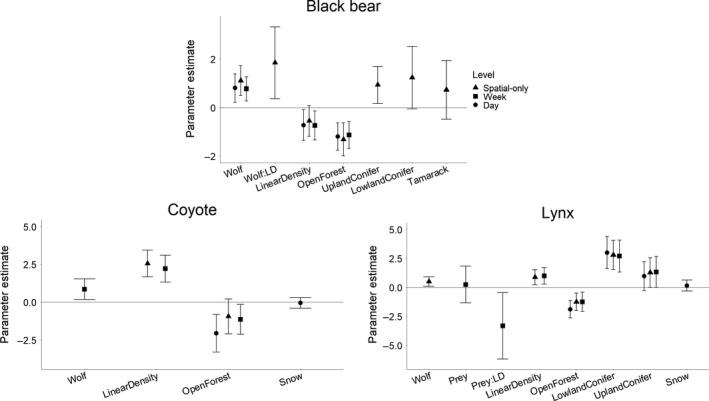
Effects of interspecific interactions and environmental features on predator occurrences. Effect sizes are shown as parameter estimates (mean ± 95% confidence intervals) from negative binomial GLMs (spatial level) and binomial GLMMs (weekly and daily levels) of black bear, coyote, and lynx occurrences at three levels of analysis. Estimates are shown for the most parsimonious model within the top‐ranked models. Estimates have not been back‐transformed, and therefore, values are not directly interpretable in terms of predator occurrences

Heterospecific occurrences were significant predictors of all three predators' occurrences, explaining variance in addition to that explained by habitat and anthropogenic features. Wolves had a positive association with all three other predators on at least one spatiotemporal scale. This effect was most consistently seen for black bears, which significantly co‐occurred with wolves at the spatial‐only, weekly, and daily scale (*β* = 1.11 [0.503, 1.722], 0.774 [0.277, 1.271], and 0.807 [0.226, 1.389], respectively; Figure 2). At the spatial‐only scale, when wolves occurred more frequently at higher linear densities, black bears also occurred more frequently (*β* = 1.850 [0.374, 3.326]). Coyotes co‐occurred with wolves at the weekly scale (*β* = 0.857 [0.175, 1.538]), but not at the spatial‐only or daily scales (Figure 2). Lynx, on the other hand, only co‐occurred with wolves at the spatial‐only scale (*β* = 0.499 [0.081, 0.918]), not on spatiotemporal scales (Figure 2).

Prey models were included in the top‐ranked models for lynx at the both the spatial‐only and weekly occurrence scales (Table 4). Lynx were less likely to occur at high linear density sites where prey were present (*β* = −1.074 [−1.934, −0.214] at the spatial‐only scale; Figure 2). Black bears and coyotes were not affected by prey occurrences at any scale (Table 4).

The occurrence of other mesocarnivores was not a strong predictor for either coyotes or lynx. For coyotes, models with lynx were included among top‐ranked models at the daily and weekly scales, but neither main effects nor season and linear density interactions were significant predictors. The same was true for lynx, although coyote occurrences only helped explain lynx occurrences at the daily scale (Table 4).

**Figure 3:**
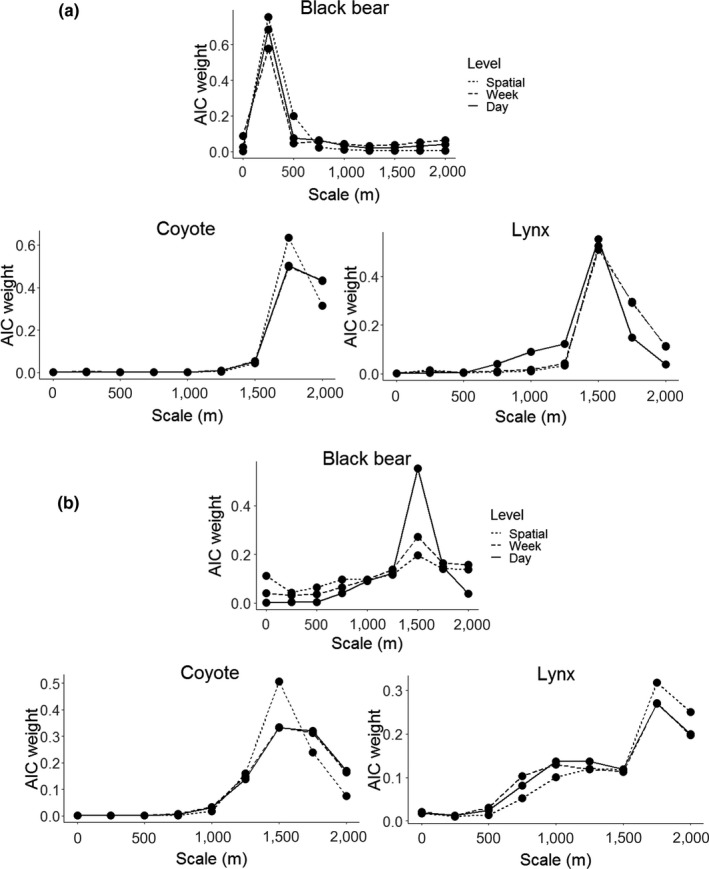
AIC model weights indicating scale of influence for habitat features (a) and linear density (b). The scale with the most model weight indicated the scale that best explains occurrences of each predator species, as determined by using AIC model selection to compare identical models measured at different spatial scales

All three predators responded to linear density on at least two scales, but the direction and scale of influence differed across species. Black bear occurrences decreased with linear density at both the weekly and the daily scales (*β* = −0.729 [−1.326, −0.131] and −0.715 [−1.347, −0.075], respectively; Figure 2). Conversely, both coyote and lynx occurrences increased with linear density at the spatial‐only and weekly scales (*β* = 2.651 [1.794, 3.507] and 2.214 [1.322, 3.106] for coyotes; 0.878 [0.229, 1.528] and 0.995 [0.283, 1.707] for lynx; Figure 2). The characteristic scale of selection for linear density was comparable for all three species (1500 m for black bears and coyotes, 1750 m for lynx) and remained constant at all three spatiotemporal scales of analysis (Figure 3b). Season was included in the top‐ranked model for both coyote and lynx at the daily scale, but did not affect occurrences for either species (Table 4).

**Figure 4:**
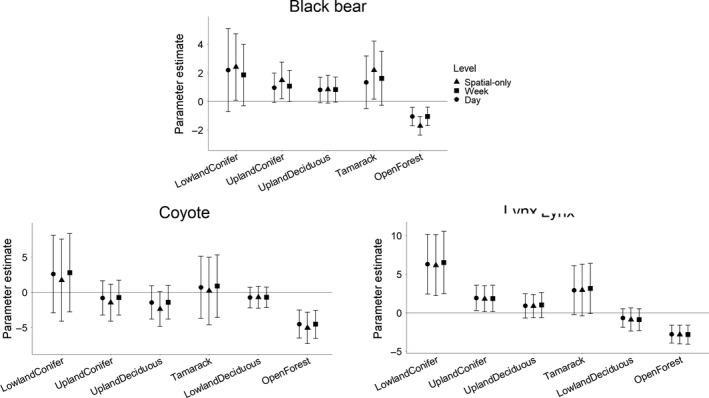
Effects of habitat features on predator occurrences in the habitat modeling step of analysis. Effect sizes are shown as parameter estimates (mean ± 95% confidence intervals) from negative binomial GLMs (spatial level) and binomial GLMMs (weekly and daily levels) of black bear, coyote, and lynx occurrences at three levels of analysis. Results are shown from habitat variables measured at the optimal spatial scale of influence: 250 m for black bears, 1750 m for coyotes, and 1500 m for lynx. Note that LowDecid is absent for black bears because lowland deciduous forest did not occur with 250 m of any camera stations. Significant habitat variables (with confidence intervals that did not overlap zero) were then included in the second step of the analysis to model effects of interspecific interactions on predators

All three predators occurred less frequently as the proportion of open forest increased on all three spatiotemporal scales of analysis (Figure 4). This significant habitat relationship was retained at all spatiotemporal scales with the inclusion of species' interactions for black bears and lynx, and at the weekly and daily scales for coyotes (Figure 2). Additionally, lynx occurrences increased with proportions of lowland and upland coniferous forest at all three scales prior to adding interspecific interactions (Figure 4). After the inclusion of occurrence variables, lynx retained the positive relationship with lowland coniferous forest at all scales and the relationship with upland coniferous forest at the spatial‐only scale (Figure 2). Black bear occurrences also increased with proportions of tamarack and lowland and upland coniferous forest at the spatial‐only scale and retained the positive relationship with upland coniferous forest with the inclusion of species' interactions (Figure 2). The characteristic scales of selection for natural habitat variables were smallest for black bears (250 m) and highest for coyotes and lynx (1750 m and 1500 m, respectively; Figure 3a). As with spatial‐only scales for linear density, the characteristic scales for each species remained constant across all spatiotemporal scales of analysis.

## 4 DISCUSSION

### 4.1 Inferring interspecific interactions from predator co‐occurrences

Black bears, coyotes, and lynx all co‐occurred with wolves on at least one of the spatial and temporal scales observed in our study (Figure 2). This suggests either that wolf spatial ecology is a determinant of space use by subordinate predators in working landscapes or that all predator species are cueing into the same resources in time and space in this complex environment (DeMars & Boutin, 2018). The poor fit of all models at the weekly and daily scales suggests that either our models do not account for other sources of variation at these finer temporal scales or our detection rates are too low to meaningfully interpret species co‐occurrences. Nevertheless, our results point toward patterns in predator use of the boreal working landscape, particularly their use of linear features. Spatiotemporal correlations among predators have potential implications for managing multipredator communities and their prey, making it crucial to understand factors underlying these relationships.

Relative to other species, wolves and black bears occur at high densities in the boreal forest (Burgar, Burton, & Fisher, 2018), and both select industrial linear features for easy travel, making them likely candidates for strong interspecific interactions in industrializing landscapes (Latham, Latham, & Boyce, 2011; Latham, Latham, Boyce, & Boutin, 2011). Although anecdotal evidence describes aggressive interactions between individual wolves and bears (Palomares & Caro, 1999; Rogers & Mech, 1981), we propose that spatiotemporal overlap at the daily scale may be consistent with facilitative interaction between the two. Black bears were more likely to occur at a site even within a day of wolf occurrences, mirroring spatiotemporal patterns in lion‐kill scavengers in Africa (Cusack et al., 2016; Swanson et al., 2016). Further, we found that black bears occurred more frequently at high linear density sites when wolves frequently occurred at those sites. Black bears are adept scavengers and may benefit considerably from carrion subsidies left by wolves (Allen et al., 2014; Wilmers et al., 2003). However, our results indicate that there are additional factors influencing black bear occurrences that were not included in our study (i.e., much unexplained variance in occurrences). Further research should investigate these sources of variance, as well as explore time elapsed between predator occurrences or analyze patterns in occurrences (i.e., which species occurs first) to observe these relationships at a finer temporal resolution (Schliep, Gelfand, Clark, & Kays, 2018; Swanson et al., 2016).

The positive association between coyotes and wolves at coarse spatiotemporal scales may also be a result of facilitation (Figure 2). Positive coyote–wolf interactions have been observed elsewhere (Atwood & Gese, 2008, 2010; Paquet, 1991, 1992; Sivy et al., 2017). However, as sympatric canid species with considerable niche overlap, coyotes and wolves are also likely to experience strong competition in which wolves frequently kill coyotes (Palomares & Caro, 1999; Paquet, 1991). We hypothesize this paradox could reflect density‐dependent interactions: Where coyotes exist in low densities, they segregate themselves from wolves to reduce competition. When coyotes exist in high densities, they may be able to reduce competition through behavioral mitigations such as increased group size or fine‐scale temporal partitioning, thus increasing the benefits of scavenging (Atwood & Gese, 2008, 2010). Coyote density (2.64/100 km^2^) eclipsed wolf density (0.77) by threefold south of our study area (Burgar et al., 2018). Densities have not yet been estimated in our study area, but wolf detections exceeded coyote detections, suggesting lower coyote relative abundance. Coyotes in this area may therefore be avoiding direct competition but benefitting indirectly via scavenging. This would be consistent with the observed co‐occurrence at the weekly scale and lack thereof at the daily scale. However, as we did not observe segregation at the daily scale and these models had substantial unexplained variance, this hypothesis requires further inquiry.

Lynx and wolves spatially co‐occurred, but did not temporally co‐occur with wolves at the weekly or daily scale (Figure 2). This suggests that although lynx share a landscape with wolves, they may not interact at finer temporal scales. We also found an unexpected negative interaction between lynx prey and linear density at the weekly scale, where lynx were less likely to occur at high linear density sites where their prey were present. This result may be a spurious result arising from low co‐occurrences between lynx and their prey (*n* = 2; Table 3). Given the poor fit of this model, we suggest that further research is needed on additional factors influencing lynx occurrences at fine temporal scales to elucidate this finding.

The negative effect of linear density on black bears, as well as the positive effect on coyotes and lynx, agrees with previous research conducted in northern Alberta (Fisher & Burton, 2018; Toews et al., 2018). We only found evidence of black bear and wolf co‐occurrences changing as a function of anthropogenic disturbance at the spatial‐only scale, indicating increasing overlap between these predators with increasing anthropogenic disturbance. This was also seen by other studies in tropical, semiurban, and mountain ecosystems (Chow‐Fraser, 2018; Karanth et al., 2017; Wang et al., 2015). This system has less of a disturbance gradient than other landscapes in Alberta's boreal forest (Government of Alberta, 2017), which might explain the lack of response from other species or at finer temporal scales. Further, humans are largely absent from the study area, so direct human influence on interspecific interactions would be minimal. To better assess the influence of anthropogenic disturbance on interspecific interactions, a similar study could be conducted across a number of landscapes with varying levels and types of landscape change (Chow‐Fraser, 2018).

An alternative interpretation of our results is that the overlapping occurrences we observed could be a result of predators responding to similar resource cues rather than responding to each other. Bears and wolves select linear features and linear feature density in similar ways (DeMars & Boutin, 2018; Finnegan et al., 2018), while coyotes and wolves may also sometimes select the same habitat (Latham et al., 2013). Like black bears and wolves, coyotes and lynx may also be using linear features as movement corridors, which may mean that all four predators prefer similar linear feature characteristics. This interpretation has important consequences for predator–prey dynamics in working landscapes, indicating high predation risk areas for prey species, particularly species at risk such as the woodland caribou (*Rangifer tarandus caribou*; DeMars & Boutin, 2018, Dickie, McNay, Sutherland, & Avgar, 2019). It would also indicate a change in functional habitat for predators, as their space use behaviors shift in response to industrial land use (Fisher & Burton, 2018). We suggest that further research into characteristics influencing linear feature use by predators would provide insight into species co‐occurrences in a working landscape.

### 4.2 Limitations

Fine‐scale temporal analyses of interspecific interactions often require large sample sizes of detections to reveal patterns in activity and co‐occurrence (Frey et al., 2017). In our study, low numbers of occurrences—and consequently, co‐occurrences—may have limited our ability to reliably model effects of interacting species (Table 3). Indeed, this is likely the cause of unexplained variance in our models at finer spatiotemporal scales. We urge caution when modeling co‐occurrences from rare or elusive species at fine spatiotemporal scales. More robust methods to assess interactions for these species could use baited camera traps to increase detection probabilities (Stewart et al., 2016), include cameras in undisturbed habitat, or increase either spatial or temporal extent of the camera trap survey to increase sampling effort. Further, telemetry studies of interacting species could account for interspecific effects on movement patterns, thereby assessing how individual animals respond to space use by other species on a shared landscape (James, Boutin, & Hebert, 2004).

Caution is obviously necessary when inferring interactions and their mechanisms from co‐occurrence data. Although techniques exist to derive interaction strength and predict mechanisms, such approaches are either nontemporal (Dorresteijn et al., 2015) or require large amounts of data (Schliep et al., 2018; Swanson et al., 2016) or even direct observation (Atwood & Gese, 2008; Cusack et al., 2016). Whereas we assume that species co‐occurrences indicate intentional proximity and thus suggest facilitative interactions, co‐occurrences of predators with similar niches may be equally indicative of species competing for a shared resource (Chow‐Fraser, 2018). To make the distinction between interaction mechanisms, spatiotemporal patterns must be related to the underlying ecological process. Camera traps offer a unique opportunity to do so by enabling direct observation of interactions while simultaneously relating this information to spatiotemporal relationships on a landscape scale (Caravaggi et al., 2017).

### 4.3 Management implications

In response to growing wolf populations, and out of concern of high predation rates on woodland caribou, the government of Alberta implements annual wolf reduction programs within some caribou herds at high risk of extirpation (Government of Alberta, 2017; Hervieux, Hebblewhite, Stepnisky, Bacon, & Boutin, 2014). Although effective in boosting caribou numbers in the short term, wolf removal is controversial and has direct consequences for the interactions structuring the boreal mammal community (Darimont, Paquet, Treves, Artelle, & Chapron, 2018; Sivy et al., 2017). As wolf reduction programs continue in caribou ranges in western Canada, we suggest that research should focus not only on caribou response, but also on responses of other species in the boreal mammal community.

Interspecific interactions arise from coexisting species partitioning space, time, and life‐sustaining resources on a shared landscape where such resources are limited (Schoener, 1974). Understanding those interactions enables us to predict how they will respond when perturbed, empowering us to make informed and proactive management decisions. Here, we showed that nonapex predators exhibit spatiotemporal overlap with an apex predator on a working landscape. This overlap identifies patterns in how these four boreal predators use this landscape, which may indicate facilitative interactions or responses to the same ecological signals. These species additionally show individual responses to anthropogenic disturbances, though responses vary and further investigation is necessary to evaluate consequences for interactions. Results from this study highlight important considerations of the impact of predator management decisions, which may unintentionally alter the behavior of coexisting species (Burgar et al., 2018). The relationships observed in this study occur in the context of a landscape experiencing ongoing industrial development, offering insight into species coexistence patterns in the face of continuing anthropogenic landscape change. To keep wildlife communities on such landscapes, we must commit to understanding the underlying relationships that allow them to thrive.

## Conflict of Interest

The authors have no competing interests to declare.

## AUTHOR CONTRIBUTIONS

ERT developed the research question and analytical framework, processed and analyzed data, and wrote the manuscript. ACB wrote the original funding proposals and developed the camera trap sampling design. JMB and ACB managed field operations, with input from JTF. ERT, JMB, JTF, and ACB acquired data, and JMB, ACB, and JTF assisted in data interpretation and provided conceptual feedback. All authors provided feedback on drafts of the manuscript.

## Data Availability

Model input data: Dryad https://doi.org/10.5061/dryad.hx3ffbg9x.
